# Towards implementing telemedicine in Tunisia: A knowledge, attitude and practice study among medical doctors

**DOI:** 10.12688/f1000research.138320.2

**Published:** 2024-08-20

**Authors:** Mariem Nouira, Nesrine Souayeh

**Affiliations:** 1 Epidemiology Department, Charles Nicolle Hospital, Faculty of Medicine of Tunis, University of Tunis El Manar, Tunis, 1006, Tunisia; 2 Gynaecology and Obstetrics Department, Regional Hospital of Ben Arous, Faculty of Medicine of Tunis, University of Tunis El Manar, Tunis, 1006, Tunisia

**Keywords:** telemedicine, knowledge, attitude, medical informatics, Tunisia

## Abstract

**Background:** The use of information and communication technology such as telemedicine occupies nowadays a huge place in modern medicine practice all over the world, mainly after the COVID-19 pandemic. However, its implementation in Tunisia and other developing countries has achieved little success with low utilization and can be challenging for several reasons. In this study, our aim was to assess the knowledge, attitudes and practice of Tunisian medical doctors regarding telemedicine.

**Methods:** This was a cross-sectional web survey, administered to medical doctors in Tunisia in October 2022. Respondents’ level of knowledge of telemedicine was assessed by calculating a knowledge score (0 to 12). Attitude subsections were about perceived telemedicine attributes of relative advantage, compatibility, trial ability and complexity.

**Results:** A total of 243 participants were included. The mean age was 45 ± 9.6 years old, and 57.2% were female, with a mean of 14.3 ± 10.3 years of professional experience. The majority (95.9%) had an average or high level of computer skills. More than half (59.3%) had a poor level of telemedicine knowledge. A good level of knowledge was significantly associated with age category over 50 years (p = 0.02) and with years of experience over 10 (p = 0.03). The majority (89.3%) had a moderate or high score about perceived advantages. The majority (88.5%) accepted use of telemedicine in their future practice. Almost half (46.9%) had practiced telemedicine activities before using a mobile phone (91%) or social media (64%). The principal limitations of applying telemedicine were challenges of organization and implementation, and incomplete patient examination.

**Conclusions:** Although Tunisian doctors’ knowledge and practice of telemedicine were unsatisfactory, their positive attitude and willingness to try it in their future practice were encouraging. There is an urgent need for implementing telemedicine in Tunisia to improve health care coverage in some unprivileged areas.

## Introduction

The world is witnessing rapid advances in audiovisual and digital technologies. The availability of the Internet has enabled impressive gains in terms of time and distance. In light of all this progress, remote medicine is developing at an increasingly rapid pace. ^1^

Information and communication technologies (ICTs) are playing an increasingly important and dominant role in the healthcare sector. They offer a multitude of solutions to the various difficulties encountered in the practice of medicine. ^2^ Telemedicine is becoming an increasingly important part of modern medical practice, in response to the new needs and challenges in the health sector. ^3^

Telemedicine is defined by the World Health Organisation as “the delivery of health-care services where distance is a critical factor, by all health-care professionals using information and communication technologies for the exchange of valid information for diagnosis, treatment and prevention of disease and injuries all in the interests of advancing the health of individuals and their communities”. ^4^

The health crisis caused by the COVID-19 pandemic highlighted the relevance and importance of telemedicine in the medical field. Greater use was made of the various forms of telemedicine during this pandemic. Telemedicine enabled equitable access to care for all socio-economic categories of patients, improved access to healthcare services for patients who were geographically isolated or had a loss of autonomy and facilitated coordination between different healthcare providers. ^5^^,^^6^

The use of ICT in the medical and health care fields is very promising in terms of improving the quality and effectiveness of medical services. ^2^ However, its implementation in Tunisia and other developing countries has achieved little success with low utilization and can be challenging for several reasons. ^5^^–^^8^

Tunisia is a North African country with a total area of approximately 163,610 square kilometers. The current population is 12,356,121 as of 2022. ^9^ The public and private sectors coexist in the structure of the Tunisian health system. Public health is a government-funded and regulated field that offers low-cost or free services to the general public. Specialized care is provided in regional and university hospitals, while primary care is provided through a network of health centers and hospitals. The doctor-to-patient ratio in Tunisia stands at approximately 1.5 doctors per 1,000 people. This ratio is slightly below the World Health Organization (WHO) recommendation of 2.3 doctors per 1,000 people for adequate healthcare delivery. ^10^ Mobile phone penetration in Tunisia is high, with a penetration rate of about 125% in 2021, meaning there are more mobile subscriptions than there are people. Mobile services are relatively affordable, contributing to the widespread use of mobile technology for communication, banking, and accessing various services. ^11^

Given all the above, the telemedicine implementation in Tunisia is needed to bridge the gap between patients and healthcare providers, especially in remote and underserved areas. Telemedicine can significantly enhance healthcare access and delivery in Tunisia.

Legal aspects of telemedicine use might be one of the major limitations of its generalization in our context. In Tunisia, the legal framework for the exercise of telemedicine has just been obtained following the publication of the presidential decree n°318/2022 in April 2022 which establishes the conditions and regulations for the practice of telemedicine in Tunisia. ^12^ This decree specifies the general conditions for conducting telemedicine activities and outlines the various fields where telemedicine can be applied. It defines the types of telemedicine services that can be provided, including remote consultations, diagnostics, and follow-up care.

In this study, our primary objective was to assess the knowledge, attitudes and practice of Tunisian doctors regarding telemedicine. Our secondary objective was to determine the obstacles to the application of telemedicine.

## Methods

The study was a cross-sectional web survey. A Google Forms questionnaire was sent by email to a large sample of doctors (approximately 5,000 email addresses) during October 2022. Theses email addresses were obtained from the Union of General Practitioners and Specialists and from the email list of university doctors from the Faculty of Medicine of Tunis. Inclusion criteria were being a Tunisian graduate doctor (generalist or specialist) practicing in Tunisia in the public or private sector and agreeing to be part of the survey.

We collected baseline demographic information and characteristics (age, gender, years of experience, speciality, computer skills …) and different questions to evaluate telemedicine knowledge, attitudes, and practice. The questionnaire was developed essentially by the authors and it was partly inspired from a study conducted in Ethiopia. ^13^ All used questions were closed questions like Yes/No, multiple choice or Likert scale questions.

### Knowledge subsection

Respondents’ level of knowledge of telemedicine was assessed by 12 questions to be answered in either “Yes” or “No.” A score of “1” was given for “Yes” and “0” for “No.” One can score a minimum of 0 and a maximum of 12 in this section. A knowledge score less than 6 was labeled as poor knowledge of telemedicine, and equal or more than a score of 6 was labeled as good knowledge of telemedicine.

### Attitude subsection

Perceived telemedicine attributes of relative advantages (7 questions), compatibility (3 questions), trial ability (7 questions) and complexity (7 questions), were rated on a four-point Likert scale that ranged from “0 = strongly disagree” to “4 = strongly agree,” except for complexity attribute questions which were reversely scored (0 = strongly agree and 4 = strongly disagree). A total mean score was calculated for each subsection of attitude questionnaire (relative advantages, compatibility, trial ability and complexity). We considered a score of each subsection of attitudes ≤49% as low, 50–70% as average, and ≥71% as high.

### Statistical analysis

For descriptive statistics, frequency and percentage values were calculated for categorical variables, and means (SD) values were used to describe continuous data.

The chi-square test was used to determine potential associations between the categorical groups. Statistical analysis was performed using SPSS software (version 23.0, IBM Corp). A p value ≤0.05 was considered as significant. Please see the underlying data. ^14^

### Ethical considerations

All participants were informed about the purpose of the study and were requested to participate in the study if they consented. All the information regarding the study was mentioned in the body text of the Google form and mail invitation. They were also informed about their right to refuse participation or drop out at any moment of the study collection process. All collected information and data analysis was confidential and anonymous during and after data collection. The approval of the ethics committee of Ben Arous Regional Hospital was obtained before conducting the study on 23 September 2022 with approval number 11/2022.

## Results

### Socio-professional characteristics

In total, 243 participants completed the questionnaire which represents a response rate = 4.9% (243/5000).

Their mean age was 45 ± 9.6 years old, and more than half (57.2%) were female, with a mean of 14.3 ± 10.3 years of professional experience. The majority (67.5%) were working in the public sector and were specialist physicians (79%) with a predominance of medical specialties (63.5%). More than half (53.9%) were hospital-university physicians. The majority worked between 31 to 50 hours per week (69.6%).

Respondents’ sociodemographic characteristics are summarized in Table 1.

**Table 1.** Socio-professional characteristics of study participants.

Socio-professional characteristics	Number	Percentage (%)
***Gender***		
Male	104	42.8
Female	139	57.2
***Age categories (years)***		
30–39	81	33.3
40–49	89	36.6
≥50	73	30
***Years of experience***		
<5	47	19.3
5–10	69	28.4
>10	127	52.3
***Practice sector***		
Public	164	67.5
Private	79	32.5
***Practice zone***		
Urban	233	95.9
Rural	10	4.1
***Specialisation***		
General medicine	51	21
Specialist	192	79
***Type of speciality***		
Medical	122	63.5
Surgical	60	31.3
Biology/fundamental	10	5.2
***Working hours/week***		
<30	17	7
31–40	95	39.1
41–50	74	30.5
51–60	29	11.9
>60	28	11.5

Most participants (95.9%) had an average to high level of computer skills. More than half of respondents (53.5%) declared that they had a good Internet connection at work and a suitable place for the practice of telemedicine (58%). For the computer tools available in the workplace, most of them had access to a computer (86.8%) but didn’t have a headset (72.4%) or camera (56.8%).

### Knowledge

Most of the respondents had heard about telemedicine (98.4%) but more than half (56.8%) didn’t know the different fields of its application.

The most well-known telemedicine activity definition was teleconsultation (63.8%).

Only 39.1% of respondents had heard about the Tunisian telemedicine decree and 25.5% (62/243) had a little knowledge of content regulation.

The knowledge score mean value was 5.2 ± 3.5 points. More than half (59.3%) had a poor level of telemedicine knowledge. A good level of knowledge was significantly associated with the age category over 50 years (p = 0.02) and with over 10 years of experience (p = 0.03) (see Table 2).

**Table 2.** Results of the association between the level of knowledge and socio-professional characteristics of the study population.

Socio-professional characteristics	Level of knowledge		p	Chi-square value (degree of freedom)
	Poor (%)	Good (%)		
***Gender***			0.13	2.207 (1)
Male	53.8	46.2		
Female	63.3	36.7		
***Age categories (years)***			0.02	7.608 (2)
30–39	67.9	32.1		
40–49	61.8	38.2		
≥50	46.6	53.4		
***Years of experience***			0.03	6.708 (2)
<5	72.3	27.7		
5–10	63.8	36.2		
>10	52	48		
***Practice zone***			1	0.002(1)
Urban	59.2	40.8		
Rural	60	40		
***Practice sector***			0.4	0.616(1)
Public	61	39		
Private	55.7	44.3		
***Specialisation***			0.3	1.067(1)
General medicine	52.9	47.1		
Specialist	60.9	39.1		
***Working hours/week***			0.07	8.421(4)
<30	52.9	47.1		
31–40	67.4	32.6		
41–50	55.4	44.6		
51–60	65.5	34.5		
>60	39.3	60.7		

The main information sources provided about telemedicine were media (TV, radio, social media) or colleagues (58.9%, N = 129 among 219 respondents).

### Attitudes

The results of doctors’ attitudes toward telemedicine are summarized in Table 3.

**Table 3.** Doctor's attitudes toward telemedicine based on perceived benefits, compatibility with their practice, ability to try, and perceived threats and disadvantages to telemedicine (N=243).

Attitude	Strongly disagree N (%)	Disagree N (%)	I Do not know (Undecided) N (%)	Agree N (%)	Strongly agree N (%)	Average score (Mean ± standard deviation)
**Benefits**						**20.1 ± 6.0**
Is an interesting practice (Useful) **for the patient**	6 **(2.5)**	11 **(4.5)**	26 **(10.7)**	88 **(36.2)**	112 **(46.1)**	
Is an interesting practice (Useful) **for the practitioner**	8 **(3.3)**	10 **(4.1)**	27 **(11.1)**	88 **(36.2)**	110 **(45.3)**	
Is an interesting practice (Useful) **for the Tunisian health system**	11 **(4.5)**	14 **(5.8)**	37 **(15.2)**	72 **(29.6)**	109 **(44.9)**	
Improves access to care	7 **(2.9)**	11 **(4.5)**	25 **(10.3)**	91 **(37.4)**	109 **(44.9)**	
Reduces the risk of medical error	41 **(16.9)**	57 **(23.5)**	71 **(29.2)**	45 **(18.5)**	29 **(11.9)**	
Facilitates diagnosis and management	15 **(6.2)**	44 **(18.1)**	63 **(25.9)**	66 **(27.2)**	55 **(22.6)**	
Facilitates communication between health professionals	5 **(2.1)**	8 **(3.3)**	20 **(8.2)**	89 **(36.6)**	121 **(49.8)**	
**Compatibility**						**7.3 ± 2.8**
Is compatible with all aspects of my clinical practice	39 **(16.0)**	57 **(23.5)**	70 **(28.8)**	39 **(16.0)**	38 **(15.6)**	
Is compatible with my current employment situation	21 **(8.6)**	32 **(13.2)**	64 **(26.3)**	67 **(27.6)**	59 **(24.3)**	
Telemedicine would be more useful for monitoring the elderly and for chronic diseases	7 **(2.9)**	12 **(4.9)**	42 **(17.3)**	100 **(41.2)**	82 **(33.7)**	
**Ability/willingness to try telemedicine**						**21.5 ± 5.0**
I wish to receive a training course in this practice	4 **(1.6)**	6 **(2.5)**	22 **(9.1)**	79 **(32.5)**	132 **(54.3)**	
It is necessary (or useful) to use telemedicine in my daily practice	7 **(2.9)**	15 **(6.2)**	31 **(12.8)**	82 **(33.7)**	108 **(44.4)**	
Trying a telemedicine application is an opportunity	4 **(1.6)**	8 **(3.3)**	26 **(10.7)**	93 **(38. 3)**	112 **(46.1)**	
Simply assessing a telemedicine application is enough to evaluate it	18 **(7.4)**	64 **(26.3)**	95 **(39.1)**	42 **(17.3)**	24 **(9.9)**	
I want to try a telemedicine application (exercise) before using it	5 **(2.1)**	5 **(2.1)**	14 **(5.8)**	105 **(43.2)**	114 **(46.9)**	
I am open (I accept) to the use of telemedicine	4 **(1.6)**	3 **(1.2)**	21 **(8.6)**	102 **(42.0)**	113 **(46.5)**	
It is necessary and useful to create a structure dedicated to the practice of telemedicine in each hospital	4 **(1.6)**	9 **(3.7)**	30 **(12.3)**	76 **(31.3)**	124 **(51.0)**	
**Threats/Complexity/Disadvantages**						**14.8 ± 4.9**
Requires too much mental effort	24 **(9.9)**	60 **(24.7)**	77 **(31.7)**	70 **(28.8)**	12 **(4.9)**	
Would be hard for me to **learn**	4 **(1.6)**	17 **(7.0)**	50 **(20.6)**	106 **(43.6)**	66 **(27.2)**	
Would be difficult for me to **apply and use**	10 **(4.1)**	19 **(7.8)**	62 **(25.5)**	98 **(40.3)**	54 **(22.2)**	
Increases workload	19 **(7.8)**	56 **(23.0)**	91 **(37.4)**	52 **(21.4)**	25 **(10.3)**	
Poses a **threat** to the practitioner's practice of medicine	14 **(5.8)**	36 **(14.8)**	87 **(35.8)**	75 **(30.9)**	31 **(12.8)**	
Engages **the medico-legal liability** of the physician who is not covered by a law that protects his rights	89 **(36.6)**	77 **(31.7)**	61 **(25.1)**	11 **(4.5)**	5 **(2.1)**	
Is a threat to patient confidentiality and privacy	29 **(11.9)**	54 **(22.2)**	81 **(33.3)**	54 **(22.2)**	25 **(10.3)**	

*Relative advantages*

The mean score of perceived relative advantages of telemedicine was 20.1 ± 6 points (ranging from 0 to 28). The majority (89.3%) had a moderate to high score relating to attitude about perceived advantages.

Most participants agreed or strongly agreed that telemedicine was useful for the patient (82.3%), for the physician (81.5%) and for the health system in general (74.5%).

Most participants agreed or strongly agreed that telemedicine improves access to health care (82.3%) and facilitates communication between healthcare professionals (86.4%).

*Compatibility*

The mean score of perceived compatibility of telemedicine was 7.3 ± 2.8 points (ranging from 0 to 12). The majority (76.5%) had a moderate to high score relating to attitude about perceived compatibility.

Only 31.7% of respondents agreed or strongly agreed that telemedicine is compatible with their clinical practice.

*Trial ability*

The mean score of perceived ability and motivation to try telemedicine (ranging between 0 to 28) was 21.5 ± 5 points. The majority (93%) had a moderate to high score relating to attitude about ability to try telemedicine.

Most of the participants agreed or strongly agreed that they would like to receive training about telemedicine (86.8 %); they thought that telemedicine is useful in their practice (78.2%) and agreed to use telemedicine in their future practice (88.5%).

*Complexity/disadvantages*

The mean score of complexity and disadvantages of telemedicine was 14.8 ± 4.9 points (ranging between 0 to 28). The majority (64.6%) had a moderate to high score relating to attitude regarding this aspect; they did not consider telemedicine as complex or having disadvantages.

The principal perceived advantages of applying telemedicine were patient interest (34.2%), tele surveillance (22.2%) and exchange of opinions between physicians (tele expertise) (20.6%).

Most of respondents thought that telemedicine is the future of medical practice (70.8%), is a necessity (72%), is a hope (70.8%) and does not interest specialists only (84%). The majority declared that they were interested in telemedicine (83.5%).

*Perceived barriers to applying telemedicine*

**Figure 1.**
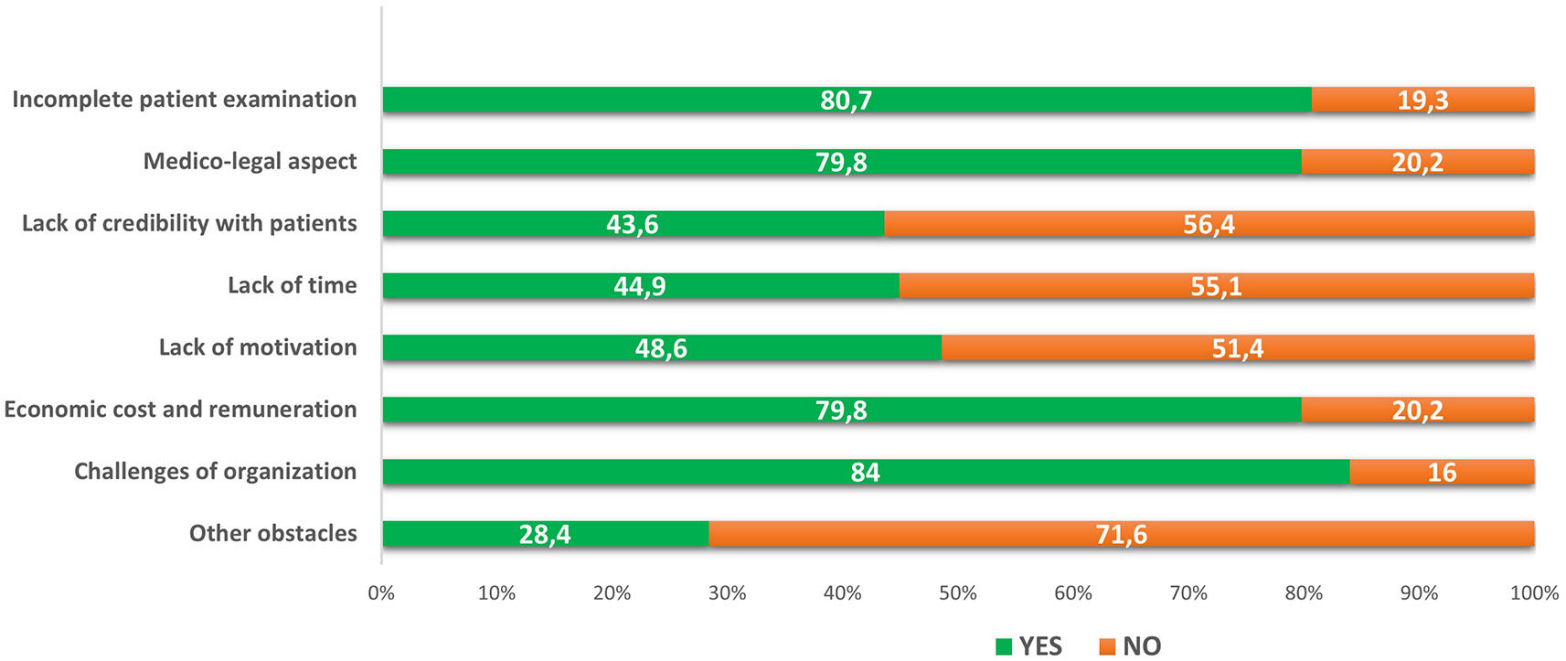
Perceived barriers to the application of telemedicine (N=243).

The principal perceived barriers to applying telemedicine were challenges of organization and implementation, incomplete patient examination, economic cost and remuneration and medico-legal aspects (see Figure 1). The practice sector (public or private) was significantly associated with challenges of organization and economic cost and remuneration barriers (p <10 ^-3^, p = 0.001, respectively). The obstacle relating to the lack of credibility with patients was found to be significantly associated with gender and age (p = 0.007, p = 0.01, respectively) (see Table 4).

**Table 4.** Identification of the main barriers, perceived by doctors, to the application of telemedicine and the study of their association with the socio-professional factors of the study population (N = 243).

Socio-professional factors	Obstacles to telemedicine implementation			
	Yes N (%)	NO N (%)	OR [CI _95%_]	p
	**Challenges of organization**			
**Gender**				0.4
Male	85 (81.7)	19 (18.3)	0.75 [0.4–1.5]	
Female	119 (85.6)	20 (14.4)		
**Age categories (years)**				0.18
30–39	72 (88.9)	9 (11.1)	-	
40–49	70 (78.7)	19 (21.3)		
≥50	62 (84.9)	11 (15.1)		
**Years of experience**				0.12
<5	44 (93.6)	3 (6.4)	-	
5–10	57 (82.6)	12 (17.4)		
>10	103 (81.1)	24 (18.9)		
**Practice sector**				**<10** ^**-3**^
Public	151 (92.1)	13 (7.9)	5.7 [2.7–11.9]	
Private	53 (67.1)	26 (32.9)		
**Practice zone**				1
Urban	195 (83.7)	38 (16.3)	0.6 [0.07–4.6]	
Rural	9 (90.0)	1 (10.0)		
**Specialisation**				0.7
General medicine	42 (82.4)	9 (17.6)	1.2 [0.5–2.6]	
Specialist	162 (84.4)	30 (15.6)		
	**Economic cost and remuneration**			
**Gender**				0.2
Male	79 (76.0)	25 (24.0)	0.6 [0.4–1.2]	
Female	115 (82.7)	24 (17.3)		
**Age categories (years)**				0.6
30–39	67 (82.7)	14 (17.3)	-	
40–49	69 (77.5)	20 (22.5)		
≥50	58 (79.5)	15 (20.5)		
**Years of experience**				0.2
<5	39 (83.0)	8 (17.0)	-	
5–10	59 (85.5)	10 (14.5)		
>10	96 (75.6)	31 (24.4)		
**Practice sector**				**0.001**
Public	141 (86.0)	23 (14.0)	3.0 [1.6–5.7]	
Private	53 (67.1)	26 (32.9)		
**Practice zone**				0.2
Urban	184 (79.0)	49 (21.0)	-	
Rural	10 (100.0)	0 (0.0)		
**Specialisation**				0.7
General medicine	40 (78.4)	11 (21.6)	1.1 [0.5–2.4]	
Specialist	154 (80.2)	38 (19.8)		
	**Lack of motivation**			
**Gender**				0.7
Male	49 (47.1)	55 (52.9)	0.9 [0.6–1.5]	
Female	69 (49.6)	70 (50.4)		
**Age categories (years)**				0.9
30–39	39 (48.1)	42 (51.9)	-	
40–49	42 (47.2)	47 (52.8)		
≥50	37 (50.7)	36 (49.3)		
**Years of experience**				0.1
<5	18 (38.3)	29 (61.7)	-	
5–10	39 (56.5)	30 (43.5)		
>10	61 (48.0)	66 (52.0)		
**Practice sector**				0.6
Public	78 (47.6)	86 (52.4)	0.9 [0.5–1.5]	
Private	40 (50.6)	39 (49.4)		
**Practice zone**				0.7
Urban	114 (48.9)	119 (51.1)	1.4 [0.4–5.2]	
Rural	4 (40.0)	6 (60.0)		
**Specialisation**				**0.04**
General medicine	31 (60.8)	20 (39.2)	0.5 [0.3–1.0]	
Specialist	87 (45.3)	105 (54.7)		
	**Lack of time**			
**Gender**				**0.02**
Male	38 (36.5)	66 (63.5)	0.6 [0.3–0.9]	
Female	71 (51.1)	68 (48.9)		
**Age categories (years)**				0.7
30–39	35 (43.2)	46 (56.8)	-	
40–49	43 (48.3)	46 (51.7)		
≥50	31 (42.5)	42 (57.5)		
**Years of experience**				0.8
<5	19 (40.4)	28 (59.6)	-	
5–10	32 (46.4)	37 (53.6)		
>10	58 (45.7)	69 (54.3)		
**Practice sector**				0.07
Public	67 (40.9)	97 (59.1)	0.6 [0.4–1.0]	
Private	42 (53.2)	37 (46.8)		
**Practice zone**				0.5
Urban	106 (45.5)	127 (54.5)	1.9 [0.5–7.7]	
Rural	3 (30.0)	7 (70.0)		
**Specialisation**				0.5
General medicine	21 (41.2)	30 (58.8)	1.2 [0.6–2.3]	
Specialist	88 (45.8)	104 (54.2)		
	**Lack of credibility with patients**			
**Gender**				**0.007**
Male	35 (33.7)	69 (66.3)	0.5 [0.3–0.8]	
Female	71 (51.1)	68 (48.9)		
**Age categories (years)**				**0.01**
30–39	45 (55.6)	36 (44.4)	-	
40–49	37 (41.6)	52 (58.4)		
≥50	24 (32.9)	49 (67.1)		
**Years of experience**				0.1
<5	24 (51.1)	23 (48.9)	-	
5–10	34 (49.3)	35 (50.7)		
>10	48 (37.8)	79 (62.2)		
**Practice sector**				0.6
Public	73 (44.5)	91 (55.5)	1.1 [0.6–1.9]	
Private	33 (41.8)	46 (58.2)		
**Practice zone**				0.1
Urban	99 (42.5)	134 (57.5)	0.3 [0.08–1.2]	
Rural	7 (70.0)	3 (30.0)		
**Specialisation**				0.2
General medicine	26 (51.0)	25 (49.0)	0.7 [0.4–1.3]	
Specialist	80 (41.7)	112 (58.3)		
	**Medico-legal aspect**			
**Gender**				0.1
Male	78 (75.0)	26 (25.0)	0.6 [0.3–1.1]	
Female	116 (83.5)	23 (16.5)		
**Age categories (years)**				0.8
30–39	66 (81.5)	15 (18.5)	-	
40–49	71 (79.8)	18 (20.2)		
≥50	57 (78.1)	16 (21.9)		
**Years of experience**				0.5
<5	40 (85.1)	7 (14.9)	-	
5–10	53 (76.8)	16 (23.2)		
>10	101 (79.5)	26 (20.5)		
**Practice sector**				**0.09**
Public	126 (76.8)	38 (23.2)	0.5 [0.3–1.2]	
Private	68 (86.1)	11 (13.9)		
**Practice zone**				0.7
Urban	186 (79.8)	47 (20.2)	0.9 [0.2–4.8]	
Rural	8 (80.0)	2 (20.0)		
**Specialisation**				0.8
General medicine	40 (78.4)	11 (21.6)	1.1 [0.5–2.4]	
Specialist	154 (80.2)	38 (19.8)		
	**Incomplete patient examination**			
**Gender**				0.2
Male	80 (76.9)	24 (23.1)	0.7 [0.3–1.2]	
Female	116 (83.5)	23 (16.5)		
**Age categories (years)**				0.2
30–39	69 (85.2)	12 (14.8)	-	
40–49	73 (82.0)	16 (18.0)		
≥50	54 (74.0)	19 (26.0)		
**Years of experience**				**0.08**
<5	42 (89.4)	5 (10.6)	-	
5–10	58 (84.1)	11 (15.9)		
>10	96 (75.6)	31 (24.4)		
**Practice sector**				0.9
Public	132 (80.5)	32 (19.5)	0.9 [0.5–1.9]	
Private	64 (81.0)	15 (19.0)		
**Practice zone**				0.1
Urban	190 (81.5)	43 (18.5)	2.9 [0.8–10.9]	
Rural	6 (60.0)	4 (40.0)		
**Specialisation**				0.6
General medicine	40 (78.4)	11 (21.6)	1.2 [0.6–2.5]	
Specialist	156 (81.3)	36 (18.8)		

### Practice

Almost half (46.9%) of interrogated physicians had practiced telemedicine activities before, using a mobile phone application (91%) or social media (64%). The majority among them (82.4%) had five years or more of professional experience; 55.3% were females; 59.6% were working in the public sector and 78.9% were specialists. There was no significant association between sociodemographic characteristics of the study population and practice of telemedicine.

A total of 63.4% declared that they intended to use telemedicine in their future activity and 32.1% were undecided.

## Discussion

Telemedicine is finding an increasingly obvious place in optimizing curative, collegial, evidence-based and local medicine in societies that are experiencing a tangible digitalization in healthcare. It is integrated as a key element in the evolution towards telehealth, which is supported by health management and integrates telecommunication systems and telecommunication technologies to protect and improve health. ^15^

The use of ICT in health care systems is likely to be influenced by many factors. The main factor appears to be the availability of an Internet connection and the necessary equipment (computer, web camera, headsets, …) as well as a suitable place for telemedicine practice. In this study, most participants declared that they had a good Internet connection (53.5%), a suitable place to perform telemedicine (58%) and access to a computer (86.8%). However, the majority did not have the required accessories. In a French study carried out in 2017 among 278 physicians, although 84% stated that they had a good Internet connection, and 99.6% had access to a computer, only 34% had appropriate premises for the practice of telemedicine, and few had accessories (36% had a camera and 25% had a headset microphone). ^16^

As for computer skills, most participants (95.9%) had an average to high level. Our findings were comparable to those of a recent study conducted in Libya, a neighbouring country, where only 26.6% of participants had professional computer skills, while 67.2% and 6.2% participants had average and beginner computer skill levels, respectively. ^17^ These skills are highly important for implementing the use of telemedicine services.

Among human-related factors influencing the use of telemedicine, components such as users’ knowledge and attitude towards technology are highly important. ^18^ Several studies have shown that attitude and perception are important and key research questions to explain how telemedicine is viewed and conceived by health professionals. ^17^^,^^19^ Our results showed that most Tunisian physicians have heard about telemedicine and the main sources of this information were media and colleagues. These findings were corroborated by the results of a large survey conducted in European countries and in Iran. ^20^

Participants’ knowledge regarding telemedicine was unsatisfactory since only 39.1% of respondents had heard about the Tunisian telemedicine decree and 59.3% had a poor level of telemedicine knowledge. Our results were consistent with studies published in developing countries. In fact, only 37.6% of medical doctors in Ethiopia had good knowledge of telemedicine. ^18^ An Egyptian study reported that only 53.6% had a good level of knowledge about telemedicine. ^21^ Moreover, a cross-sectional study in India found similar results (41% had good knowledge). ^22^ The level of telemedicine knowledge in developing countries remains poor due to several factors. In many developing countries, healthcare professionals have limited awareness and education about telemedicine. This is partly because telemedicine is not yet widely used and there is a lack of training and educational programs to inform healthcare professionals about its benefits and applications. Also, the lack of clear regulations and policies for telemedicine in these countries contributes to a low level of knowledge.

A good level of knowledge was significantly associated with the age category over 50 years (p = 0.02) and with years of experience over 10 (p = 0.03) in our study. These results were different from most studies. In fact, a cross-sectional Indian survey observed higher knowledge scores among MDs younger than 50-years-old. ^20^ Moreover, Barton *et al.* found a significant difference in self-assessed knowledge and beliefs about telemedicine between the specialist physicians who were users of telemedicine and specialist physicians who were non-users of telemedicine. ^23^

Most of the participants in our survey had a positive attitude towards telemedicine and its perceived advantages. Similar results were found among healthcare providers in Egypt, Saudi Arabia and Ethiopia. ^13^^,^^21^^,^^24^ Even with limited knowledge, healthcare providers in developing countries often perceive telemedicine positively due to its potential to improve access to healthcare, especially in remote and underserved areas. They see it as a tool that can help bridge the gap in healthcare delivery. A survey in Michigan State University, USA, and other similar studies showed that the attitude of health care workers is an important factor in understanding and accepting telemedicine technologies. ^18^^,^^22^^,^^25^^–^^27^ These facts are important, especially in countries struggling with implementing telemedicine in their routine practice, because attitude represents how telemedicine is perceived by health care workers. For such acceptance, program developers need to train health care workers and make the telemedicine programs usable for them. ^28^

Furthermore, the most cited advantages of telemedicine by Tunisian physicians were improving access to health care (82.3%) and facilitating communication between healthcare professionals (86.4%). The benefits of telemedicine were widely discussed in the literature, among them we found: promoting cooperation between public and private fields, reducing the waste of time and long trips for patients, resolving medical desert issues, treating isolated patients or those who cannot travel, adapting the supply of care to demographic changes and meeting the needs related to demographic changes and finally being able to respond to the growing number of patients suffering from chronic diseases. ^29^^–^^31^ Moreover, qualitative studies emphasised the importance of economic gain when using telemedicine. ^29^^,^^30^

Most of our participants expressed their ability to try telemedicine in their future practice. In a large cross-sectional conducted in India, only 60% expressed interest in adopting this new technology in their future career. ^20^ However, Ethiopian physicians were more open to trying telemedicine since 93.3% of them agreed or strongly agreed that trying telemedicine was a great opportunity and 81.9% of them would like to begin a telemedicine application. ^6^

The majority of interrogated Tunisian doctors found telemedicine not complex and compatible with most of their practice aspects (64.6%). However, they expressed some serious concerns regarding the challenges of organization and implementation (84%), incomplete patient examination (80.7%), economic cost and remuneration (79.8%) and medico-legal aspects (79.8%). Moreover, only 31.7% thought that telemedicine was compatible with their clinical practice. The same concerns were expressed in other studies. ^6^^,^^22^ Furthermore, new ethical issues have emerged from this type of medical practice regarding patients’ confidentiality. ^28^ These findings indicate that much work is needed to be done to educate health care professionals about telemedicine and to lay the groundwork for successful and sustainable adoption of the technology in the country. ^6^

Moreover, other limitations to the implementation of telemedicine in resource limited countries deserve to be mentioned, such as technical issues and defective health care infrastructure restricting the potential for swift and innovative reforms. ^32^^–^^34^

As for practicing telemedicine, 46.9% of interrogated physicians had practiced telemedicine activities before, using a mobile phone (91%) or social media (64%). This emphasises the need for creating a suitable common platform in order to properly practice telemedicine and therefore avoid some confidentiality issues relating to the use of the phone or social media.

### Strengths and limitations of our study

This was the first Tunisian publication studying knowledge, attitudes, and practice of telemedicine among Tunisian practitioners. Our sample size was considerable, and we included all kinds of MD (public and private, specialist and non-specialist physicians). However, the study sample was not representative of all medical practitioners and we did not take into consideration cultural aspects relating to telemedicine, or the patient’s perspective on the issue.

### Recommendations

We recommend establishing continuous professional development programs to enhance healthcare professionals' knowledge of telemedicine, including its legislation in Tunisia, and to provide training in software and computer skills. Additionally, training in ethical and medico-legal issues related to telemedicine is crucial to ensure patient confidentiality.

For future perspectives, we propose a comprehensive study of telemedicine in greater depth and gather the perspectives of different stakeholders, including policymakers, platform managers, doctors, and patients. Our study addressed the issue of telemedicine from the perspective of doctors only. Further studies should target patients and policymakers to understand their perceptions of telemedicine, aiming for a comprehensive vision that includes the opinions of all stakeholders.

Moreover, qualitative studies could be valuable to gather expert opinions on the benefits and challenges of adopting telemedicine in the medical field. Understanding different practical implementation modalities before initiating large-scale projects can help ensure their success and profitability.

## Conclusions

In conclusion, although Tunisian physicians’ knowledge level and practice of telemedicine were unsatisfactory, their positive attitude and willingness to try it in their future practice was encouraging. The Tunisian government should encourage this practice by must create the enabling environment, develop strategy and policies for telemedicine, and set up regulations.

## Consent

Written informed consent for publication of the participants’ details was obtained from the participants.

## Data Availability

Harvard Dataverse: Underlying data for ‘Toward implementing telemedicine in Tunisia: Results of a knowledge, attitude and practice study among medical doctors’, “Telemedicine-Tunisia”, https://doi.org/10.7910/DVN/AAMOQN. ^14^ This project contains the following underlying data:
• Date file 1: Telemedicine-Tunisia.xlsx (anonymised underlying data collected from medical doctors) Harvard Dataverse: Extended data for ‘Toward implementing telemedicine in Tunisia: Results of a knowledge, attitude and practice study among medical doctors’, “Telemedicine-Tunisia”, https://doi.org/10.7910/DVN/AAMOQN. ^14^ This project contains the following extended data:
• Questionnaire: Telemedicine – English version.pdf Data are available under the terms of the Creative Commons Zero “No rights reserved” data waiver (CC0 1.0 Public domain dedication).
