# Supplementary material for: Towards implementing telemedicine in Tunisia: A knowledge, attitude and practice study among medical doctors
Source: F1000Res. 2024 Aug 20;12:1025. Originally published 2023 Aug 22. [Version 2] doi: 10.12688/f1000research.138320.2 (PMC11377923; doi:10.12688/f1000research.138320.2)
Supplement: Peer review history

# Reviewer report: Reviewer response for version 2

**DOI:** 10.5256/f1000research.170105.r315506

**Published:** 2024-09-30

**Authors:** Sumaiah Alrawiai

**Affiliations:** 1 Imam Abdulrahman Bin Faisal University, Dammam, Eastern Province, Saudi Arabia

The authors have addressed my concerns. However, in the future, I suggest you refer to the exact page and line where you made the requested modification, to make it easier for the reviewer to view the changes made.

Is the work clearly and accurately presented and does it cite the current literature?

Yes

If applicable, is the statistical analysis and its interpretation appropriate?

I cannot comment. A qualified statistician is required.

Are all the source data underlying the results available to ensure full reproducibility?

Yes

Is the study design appropriate and is the work technically sound?

Yes

Are the conclusions drawn adequately supported by the results?

Yes

Are sufficient details of methods and analysis provided to allow replication by others?

Partly

Reviewer Expertise:

My research topics include patient-centered care, shared decision-making, and patient-doctor communication. I've done some studies in digital health as well.

I confirm that I have read this submission and believe that I have an appropriate level of expertise to confirm that it is of an acceptable scientific standard.

# Reviewer report: Reviewer response for version 2

**DOI:** 10.5256/f1000research.170105.r318542

**Published:** 2024-09-05

**Authors:** Giulio Nittari

**Affiliations:** 1 School of Medicinal and Health Products Sciences, University of Camerino, Camerino, Italy

A good analysis of the topic regarding telemedicine use in Tunisia. 

 some modifications are required before accepting for indexing:

 In the introduction section/ or discussion a thorough analysis of telemedicine and telehealth should be mentioned in regards to and especially after covid-19, since more and more health professionals tend to telemedicine for patient evaluation and follow-up during this period and most of them implement it still nowadays. 

 you can draw information from:

 (Ricci et al., 2020)(Ref-1)

 (Nittari et al.,2022)(Ref-2)

 In discussion section a comparison regarding European countries should be noted to have a more overall complete discussion of the topic: Information can be drawn from:

 (Ricci et al., 2020)(Ref-1)

 In limitations and recommendations a mention is done regarding patients and patience compliance regarding telemedicine as well as medicolegal implications need further analysis. Information regarding these two aspects can be found in:

 (Nittari et al.,2023)(Ref-3)

Is the work clearly and accurately presented and does it cite the current literature?

Partly

If applicable, is the statistical analysis and its interpretation appropriate?

Yes

Are all the source data underlying the results available to ensure full reproducibility?

Yes

Is the study design appropriate and is the work technically sound?

Yes

Are the conclusions drawn adequately supported by the results?

Partly

Are sufficient details of methods and analysis provided to allow replication by others?

Partly

Reviewer Expertise:

Telemedicine, Telepharmacy, Neuroimmaging, Neurodegeneration, neuroinflammation, Public health

I confirm that I have read this submission and believe that I have an appropriate level of expertise to confirm that it is of an acceptable scientific standard, however I have significant reservations, as outlined above.

## References

[1] : Consequences of COVID-19 Outbreak in Italy: Medical Responsibilities and Governmental Measures. *Front Public Health* .2020;8: 10.3389/fpubh.2020.588852 588852 10.3389/fpubh.2020.588852 33364225 PMC7752765

[2] : Telemedicine in the COVID-19 Era: A Narrative Review Based on Current Evidence. *Int J Environ Res Public Health* .2022;19(9) : 10.3390/ijerph19095101 10.3390/ijerph19095101 35564494 PMC9105428

[3] : Telemedicine in diabetic ulcer management: A pilot study with exploration of medico-legal aspects. *Nutr Metab Cardiovasc Dis* .2023;33(11) : 10.1016/j.numecd.2023.07.021 2280-2286 10.1016/j.numecd.2023.07.021 37591741

# Reviewer report: Reviewer response for version 2

**DOI:** 10.5256/f1000research.170105.r315507

**Published:** 2024-08-27

**Authors:** Benson Ncube

**Affiliations:** 1 University of KwaZulu-Natal, Durban, South Africa

The authors have satisfactorily addressed my concerns in their manusrcipt.

 However, they need to replace "by" with "but" in their last sentence in the conclusion section.

Is the work clearly and accurately presented and does it cite the current literature?

Yes

If applicable, is the statistical analysis and its interpretation appropriate?

Partly

Are all the source data underlying the results available to ensure full reproducibility?

Partly

Is the study design appropriate and is the work technically sound?

Partly

Are the conclusions drawn adequately supported by the results?

Partly

Are sufficient details of methods and analysis provided to allow replication by others?

Partly

Reviewer Expertise:

Digital health strategy and initiatives

I confirm that I have read this submission and believe that I have an appropriate level of expertise to confirm that it is of an acceptable scientific standard.

# Reviewer report: Reviewer response for version 1

**DOI:** 10.5256/f1000research.151510.r281821

**Published:** 2024-06-26

**Authors:** Sumaiah Alrawiai

**Affiliations:** 1 Imam Abdulrahman Bin Faisal University, Dammam, Eastern Province, Saudi Arabia

This is an interesting paper that aims to explore the knowledge, attitudes, and practice of telemedicine by doctors in Tunisia.

 However, there are some issues that need to be addressed and they are presented here:

1. The introduction could benefit from additional information on the current healthcare landscape in Tunisia and how telemedicine could help. What are the possible barriers and facilitators to adopting telemedicine in Tunisia, or if not available, countries with similar healthcare system to Tunisia?
2. Towards the end of the introduction, the objective was stated but it was missing the practice part. Only knowledge and attitudes were mentioned.
3. The methods section is missing some information. How did you develop the questionnaire? Was it adopted from another study, or was it developed by the authors? If developed by authors, how did you develop it, and what were the sources used? If it was adopted from another study, then please reference the source.
4. Also, in the methods section, when sending the invites, did you send any reminders, and were there any differences between those who responded and those who didn’t? Was the sample size enough? How did you ensure that?
5. If the questionnaire was developed by the authors, how did you ensure the validity and reliability of the questionnaire?
6. Under the discussion, some areas would benefit from an in-depth analysis of why there was a difference or a similarity between your study and other studies.

Is the work clearly and accurately presented and does it cite the current literature?

Yes

If applicable, is the statistical analysis and its interpretation appropriate?

I cannot comment. A qualified statistician is required.

Are all the source data underlying the results available to ensure full reproducibility?

Yes

Is the study design appropriate and is the work technically sound?

Yes

Are the conclusions drawn adequately supported by the results?

Yes

Are sufficient details of methods and analysis provided to allow replication by others?

Partly

Reviewer Expertise:

My research topics include patient-centered care, shared decision-making, and patient-doctor communication. I've done some studies in digital health as well.

I confirm that I have read this submission and believe that I have an appropriate level of expertise to confirm that it is of an acceptable scientific standard, however I have significant reservations, as outlined above.

# Response

**Published:** 2024-08-06

**Authors:** Mariem Nouira

**Affiliations:** Faculty Of Medicine Of Tunis-Tunisia, Tunisia

Dear Dr Sumaiah Alrawiai,

 Thank you very much for your review. We really appreciate your response and time. We have revised our manuscript as you suggested. Here are the responses to your comments:

**Reviewer comment: **The introduction could benefit from additional information on the current healthcare landscape in Tunisia and how telemedicine could help. What are the possible barriers and facilitators to adopting telemedicine in Tunisia, or if not available, countries with similar healthcare system to Tunisia?

**Author response: **The introduction was modified. For the barriers to adopting telemedicine in Tunisia, it was the secondary objective of our study, and it was responded in the result section. It was also discussed in the discussion. Thank you.

**Reviewer comment: **Towards the end of the introduction, the objective was stated but it was missing the practice part. Only knowledge and attitudes were mentioned.

**Author response: **It was corrected. Thank you

**Reviewer comment: **The methods section is missing some information. How did you develop the questionnaire? Was it adopted from another study, or was it developed by the authors? If developed by authors, how did you develop it, and what were the sources used? If it was adopted from another study, then please reference the source.

**Author response: **The questionnaire was developed essentially by the authors, and it was partly inspired from a study conducted in Ethiopia that was cited. Thank you.

**Reviewer comment: **Also, in the methods section, when sending the invites, did you send any reminders, and were there any differences between those who responded and those who didn’t? Was the sample size enough? How did you ensure that?

**Author response: **We did not send reminders. And we didn’t have information about the non responders so we can’t compare them with the responders. We didn’t calculate the sample size because we didn’t have the results of a Tunisian study that was conducted previously using the same questionnaire to use the parameters to calculate the sample size. Also, we didn’t find in the literature a specific formula to calculate sample size of Knowledge Attitude and Practice studies.

**Reviewer comment: **If the questionnaire was developed by the authors, how did you ensure the validity and reliability of the questionnaire?

**Author response: **The reliability and validity of the questionnaire was not studied in our manuscript. It could constitute the aim of another manuscript. Thank you.

**Reviewer comment: **Under the discussion, some areas would benefit from an in-depth analysis of why there was a difference or a similarity between your study and other studies.

**Author response: **The discussion was enhanced and modified. Thank you very much.

# Reviewer report: Reviewer response for version 1

**DOI:** 10.5256/f1000research.151510.r257775

**Published:** 2024-05-07

**Authors:** Benson Ncube

**Affiliations:** 1 University of KwaZulu-Natal, Durban, South Africa

1. **General,**

 The authors are discussing an important aspect in the field of telemedicine. This is a quite relevant topic and they are commended for their work. Albeit some comments are presented to redeem this manuscript.

 2. In the topic, I suggest the authors consider deleting the words "Results of a", because we know they are presenting the results.

 3. **Abstract.** The sample size must be stated in this section.

 4. **Introduction.** What about "cloud and mobile computing?"In para 2, The use of ICTs is not only dominant in the health sector.  "Telemedicine, one of the most promising forms of ICT," This is not a true statement. Rephrase.

 In para 3, I suggest you use the current definition of telemedicine by WHO. In para 5, please cite more African cases, eg. Botswana, South Africa, Egypt, Lybia, etc. There are plenty of such cases. A little bit of explanation of the presidential decree will give a better understanding to the reader.

 Authors are requested to provide a more detailed context on where the study took place. What is the land size of Tunisia? population of the general public? how is the health system structured in Tunisia? What is the doctor/patient ratio? Compare this ratio with the recommended WHO ratio for Tunisia. How is the mobile phone penetraition rate, and the affordability of mobile phone services?

 5. **Methods.** I suggest authors give a detailed description of the Questionnaire, in terms of types and number of questions, e.g. Likert, dichotomous, open-ended, closed questions. What is the sample size? Authors should be very clear on this, as they know exactly how many participants they sampled.  Determine and present the response rate of participants. Your design of the study needs to be clearly articulated, to cover the sample size, sampling strategy, inclusion/exclusion criteria, and data analysis methods used. Discuss the use of statistics in the design. The results of the analysis should be presented in the Results section.

 There is a need to present your Chi-square Test results, in order to help the reader to understand your presented results.

 6. **Results.** In Table 2, why is it that some p-values are not presented? Inclusion of all p-values will make your table more complete. On page 5, where authors report statistically significant results, they should provide the statistics (Chi-square value), degree of freedom for the contigence table together with the p-value. This is the proper way of reporting this kind of result.

 In Fig 1, you use the word  "Main", which are the minor barriers then?

 When reporting under the sub-heading  "Perceived barriers ...." you used percentages which is a repetition of results since they are already reported in Fig. 1.

 In sub-topic "Practice" you need to use items in the same class" So in the first sentence, you cannot use "mobile phone" but "mobile phone application" 

 7. **Discussion. **In the first paragraph, I suggest you add "in healthcare" at the end of the first sentence. Replace "our" with "this" as we know it is your study. It would appear there is an over use of the word "declared" throughout the paper. Please find alternatives. In the third paragraph replace were with where. In this paragraph, it will be interesting to compare your results with those of other countries like Botswana, Egypt, Nigeria, Pakistan, Ethiopia. (Ref-1). Do the same in paragraphs 4 & 5. In the subsequent paragraphs, I suggest you extract all your recommendations and put them under a sub-topic  "Recommendations".  

 In paragraph 3, what about referrals, access to special services, and seeking or giving second opinion? You did not consider COVID-19 aspects? In paragraph 6, is the term "limited resources countries" or "resource limited countries"? In paragraph 7, an ambiguous clause is noted - using a mobile phone (91%) or social media (64%).

 In the section, limitations of the study, what about the following? You did not consider the diversity of healthcare workers, those without access to the Internet.

 8. **Conclusion. **Should the government provide equipment and telemedicine platforms? Or must create the enabling environment, develop strategy and policies for telemedicine, and set up regulations?

Is the work clearly and accurately presented and does it cite the current literature?

Yes

If applicable, is the statistical analysis and its interpretation appropriate?

Partly

Are all the source data underlying the results available to ensure full reproducibility?

Partly

Is the study design appropriate and is the work technically sound?

Partly

Are the conclusions drawn adequately supported by the results?

Partly

Are sufficient details of methods and analysis provided to allow replication by others?

Partly

Reviewer Expertise:

Digital health strategy and initiatives

I confirm that I have read this submission and believe that I have an appropriate level of expertise to confirm that it is of an acceptable scientific standard, however I have significant reservations, as outlined above.

## References

[1] : Perceptions and attitudes of patients and healthcare workers towards the use of telemedicine in Botswana: An exploratory study. *PLoS One* .2023;18(2) : 10.1371/journal.pone.0281754 e0281754 10.1371/journal.pone.0281754 36795740 PMC9934446

# Response

**Published:** 2024-08-06

**Authors:** Mariem Nouira

**Affiliations:** Faculty Of Medicine Of Tunis-Tunisia, Tunisia

Dear Dr Benson Ncube,

 Thank you very much for your review. We really appreciate your response and time. We have revised our manuscript as you suggested. Here are the responses to your comments:

**Reviewer comment:** In the topic, I suggest the authors consider deleting the words "Results of a", because we know they are presenting the results.

**Author response:** It was corrected. Thank you

 “Towards implementing telemedicine in Tunisia: A knowledge, attitude and practice study among medical doctors”

**Reviewer comment: **Abstract. The sample size must be stated in this section.

**Author response: **We didn’t calculate the sample size because we didn’t have the results of a Tunisian study that was conducted previously using the same questionnaire to use the parameters to calculate the sample size. Also, we didn’t find in the literature a specific formula to calculate sample size of Knowledge Attitude and Practice studies. 

**Reviewer comment:** Introduction. What about "cloud and mobile computing?"In para 2, The use of ICTs is not only dominant in the health sector.  "Telemedicine, one of the most promising forms of ICT," This is not a true statement. Rephrase. In para 3, I suggest you use the current definition of telemedicine by WHO. In para 5, please cite more African cases, eg. Botswana, South Africa, Egypt, Lybia, etc. There are plenty of such cases. A little bit of explanation of the presidential decree will give a better understanding to the reader.Authors are requested to provide a more detailed context on where the study took place. What is the land size of Tunisia? population of the general public? how is the health system structured in Tunisia? What is the doctor/patient ratio? Compare this ratio with the recommended WHO ratio for Tunisia. How is the mobile phone penetraition rate, and the affordability of mobile phone services?

**Author response:** All suggested modifications were done thank you.

**Reviewer comment: **Methods. I suggest authors give a detailed description of the Questionnaire, in terms of types and number of questions, e.g. Likert, dichotomous, open-ended, closed questions. 

**Author response: **All suggested modifications were done thank you.

**Reviewer comment:** Results. In Table 2, why is it that some p-values are not presented? Inclusion of all p-values will make your table more complete. On page 5, where authors report statistically significant results, they should provide the statistics (Chi-square value), degree of freedom for the contigence table together with the p-value. This is the proper way of reporting this kind of result.

**Author response: **The p-values of all variables (one p for each variable) were presented in the table 2. All other suggested modifications were done. Thank you.

**Reviewer comment: **In Fig 1, you use the word  "Main", which are the minor barriers then?When reporting under the sub-heading  "Perceived barriers ...." you used percentages which is a repetition of results since they are already reported in Fig. 1. In sub-topic "Practice" you need to use items in the same class" So in the first sentence, you cannot use "mobile phone" but "mobile phone application"

**Author response:** All suggested modifications were done thank you. 

**Reviewer comment:** Discussion. In the first paragraph, I suggest you add "in healthcare" at the end of the first sentence. Replace "our" with "this" as we know it is your study. It would appear there is an over use of the word "declared" throughout the paper. Please find alternatives. In the third paragraph replace were with where. In this paragraph, it will be interesting to compare your results with those of other countries like Botswana, Egypt, Nigeria, Pakistan, Ethiopia. (Ref-1). Do the same in paragraphs 4 & 5. In the subsequent paragraphs, I suggest you extract all your recommendations and put them under a sub-topic  "Recommendations".  

**Author response:** All suggested modifications were done thank you. 

**Reviewer comment:**  In the section, limitations of the study, what about the following? You did not consider the diversity of healthcare workers, those without access to the Internet.

**Author response:** All suggested modifications were done thank you. 

**Reviewer comment: ** Conclusion. Should the government provide equipment and telemedicine platforms? Or must create the enabling environment, develop strategy and policies for telemedicine, and set up regulations?

**Author response:** All suggested modifications were done thank you very much.
