# Supplementary material for: Towards implementing telemedicine in Tunisia: Results of a knowledge, attitude and practice study among medical doctors
Source: F1000Res. 2023 Aug 22;12:1025. [Version 1] doi: 10.12688/f1000research.138320.1 (PMC11377923; doi:10.12688/f1000research.138320.1)
Supplement: Peer review history

# Response

**Published:** 2024-08-06

**Authors:** Mariem Nouira

**Affiliations:** Faculty Of Medicine Of Tunis-Tunisia, Tunisia

Dear Dr Benson Ncube,

 Thank you very much for your review. We really appreciate your response and time. We have revised our manuscript as you suggested. Here are the responses to your comments:

**Reviewer comment:** In the topic, I suggest the authors consider deleting the words "Results of a", because we know they are presenting the results.

**Author response:** It was corrected. Thank you

 “Towards implementing telemedicine in Tunisia: A knowledge, attitude and practice study among medical doctors”

**Reviewer comment: **Abstract. The sample size must be stated in this section.

**Author response: **We didn’t calculate the sample size because we didn’t have the results of a Tunisian study that was conducted previously using the same questionnaire to use the parameters to calculate the sample size. Also, we didn’t find in the literature a specific formula to calculate sample size of Knowledge Attitude and Practice studies. 

**Reviewer comment:** Introduction. What about "cloud and mobile computing?"In para 2, The use of ICTs is not only dominant in the health sector.  "Telemedicine, one of the most promising forms of ICT," This is not a true statement. Rephrase. In para 3, I suggest you use the current definition of telemedicine by WHO. In para 5, please cite more African cases, eg. Botswana, South Africa, Egypt, Lybia, etc. There are plenty of such cases. A little bit of explanation of the presidential decree will give a better understanding to the reader.Authors are requested to provide a more detailed context on where the study took place. What is the land size of Tunisia? population of the general public? how is the health system structured in Tunisia? What is the doctor/patient ratio? Compare this ratio with the recommended WHO ratio for Tunisia. How is the mobile phone penetraition rate, and the affordability of mobile phone services?

**Author response:** All suggested modifications were done thank you. 

     

**Reviewer comment: **Methods. I suggest authors give a detailed description of the Questionnaire, in terms of types and number of questions, e.g. Likert, dichotomous, open-ended, closed questions. 

**Author response: **All suggested modifications were done thank you. 

**Reviewer comment:** Results. In Table 2, why is it that some p-values are not presented? Inclusion of all p-values will make your table more complete. On page 5, where authors report statistically significant results, they should provide the statistics (Chi-square value), degree of freedom for the contigence table together with the p-value. This is the proper way of reporting this kind of result.

**Author response: **The p-values of all variables (one p for each variable) were presented in the table 2. All other suggested modifications were done. Thank you.

**Reviewer comment: **In Fig 1, you use the word  "Main", which are the minor barriers then?When reporting under the sub-heading  "Perceived barriers ...." you used percentages which is a repetition of results since they are already reported in Fig. 1. In sub-topic "Practice" you need to use items in the same class" So in the first sentence, you cannot use "mobile phone" but "mobile phone application"

**Author response:** All suggested modifications were done thank you. 

**Reviewer comment:** Discussion. In the first paragraph, I suggest you add "in healthcare" at the end of the first sentence. Replace "our" with "this" as we know it is your study. It would appear there is an over use of the word "declared" throughout the paper. Please find alternatives. In the third paragraph replace were with where. In this paragraph, it will be interesting to compare your results with those of other countries like Botswana, Egypt, Nigeria, Pakistan, Ethiopia. (Ref-1). Do the same in paragraphs 4 & 5. In the subsequent paragraphs, I suggest you extract all your recommendations and put them under a sub-topic  "Recommendations".  

**Author response:** All suggested modifications were done thank you. 

**Reviewer comment:**  In the section, limitations of the study, what about the following? You did not consider the diversity of healthcare workers, those without access to the Internet.

**Author response:** All suggested modifications were done thank you. 

**Reviewer comment: ** Conclusion. Should the government provide equipment and telemedicine platforms? Or must create the enabling environment, develop strategy and policies for telemedicine, and set up regulations?

**Author response:** All suggested modifications were done thank you very much.
